# The Methyltransferases PRMT4/CARM1 and PRMT5 Control Differentially Myogenesis in Zebrafish

**DOI:** 10.1371/journal.pone.0025427

**Published:** 2011-10-10

**Authors:** Julie Batut, Carine Duboé, Laurence Vandel

**Affiliations:** 1 Université de Toulouse - Paul Sabatier, Centre de Biologie du Développement, Toulouse, France; 2 Centre National de la Recherche Scientifique, Centre de Biologie du Développement, UMR 5547, Toulouse, France; Ecole Normale Supérieure de Lyon, France

## Abstract

In vertebrates, skeletal myogenesis involves the sequential activation of myogenic factors to lead ultimately to the differentiation into slow and fast muscle fibers. How transcriptional co-regulators such as arginine methyltransferases PRMT4/CARM1 and PRMT5 control myogenesis *in vivo* remains poorly understood. Loss-of-function experiments using morpholinos against PRMT4/CARM1 and PRMT5 combined with *in situ* hybridization, quantitative polymerase chain reaction, as well as immunohistochemistry indicate a positive, but differential, role of these enzymes during myogenesis *in vivo*. While PRMT5 regulates *myod*, *myf5* and *myogenin* expression and thereby slow and fast fiber formation, PRMT4/CARM1 regulates *myogenin* expression, fast fiber formation and does not affect slow fiber formation. However, our results show that PRMT4/CARM1 is required for proper slow myosin heavy chain localization. Altogether, our results reveal a combinatorial role of PRMT4/CARM1 and PRMT5 for proper myogenesis in zebrafish.

## Introduction

The proper control of gene expression is essential for normal growth, development and differentiation. An early step in the process of gene activation consists in modifying histones and unraveling DNA sequences within promoter regions thereby facilitating the recruitment of specific and general transcription factors. Skeletal muscle differentiation involves cooperation between the muscle regulatory factors (MRFs) Myod, Myf5, Mrf4 and Myogenin [1], members of the Myocyte-enhancer factors 2 (Mef2) family and chromatin remodelling or modifying enzymes [2]. Numerous histone modifying enzymes have been implicated in the regulation of myogenic genes in cell culture including members of protein arginine methyltransferase (PRMT)- families [2]. Of particular interest are two members of the PRMT family, PRMT4/CARM1 (hereafter, called CARM1) and PRMT5 as independent studies performed in cell culture or on dissected skeletal tissues suggested that they are required for myogenesis [3], [4], [5]. CARM1 methylates histone H3 on specific arginine residues to regulate transcription [6], [7], as well as non-histone proteins such as the transcriptional co-activators CBP/p300 [8], [9], [10], [11]. Over the last few years, several studies have shown that CARM1 acts as transcriptional co-activator in cell proliferation and in a multitude of signaling pathways [12], [13]. Similarly, PRMT5 methylates histones H3 and H4 as well as non-histone proteins but has been shown to regulate negatively cell proliferation [13], [14]. These data suggest that CARM1 and PRMT5 play a central role in muscle differentiation in cell culture and most likely *in vivo*. However, the mechanisms by which these enzymes control myogenesis have not been addressed yet. Indeed, no other report has addressed the precise role(s) of CARM1 and PRMT5 *in vivo* during development and particularly in myogenesis. To gain insights into their function *in vivo*, we turned to zebrafish to analyze their shared and divergent roles in myogenesis.

In zebrafish, cells are committed to the myogenic fate through the activation and the cooperation of MRFs and Mef2 proteins [15]. Then, committed myoblasts differentiate into muscles fibers with distinct genes expression profiles and contraction speeds, the so-called slow- and fast-twitch fibers. In the zebrafish embryo, progenitors of the slow and fast muscle fibers can be identified on the basis of their morphology, their position within the segmental plate before somites formation and the expression of a specific set of genes [16]. It has been shown recently that *myf5* and *myod*, but not *myogenin*, are required and cooperate to drive myoblast progenitor (also called adaxial cells) commitment to the slow fiber type [17] whereas fast fiber differentiation occurs during the migration of slow fibers and relies on the expression of *myf5* and *myod* but also of *myogenin* [17], [18]. Here, we investigated the involvement of CARM1 and PRMT5 in myogenesis in zebrafish embryos. We found that both CARM1 and PRMT5 control *myogenin* expression whereas PRMT5, controls the expression of the early genes *myod* and *myf5*. Accordingly, we demonstrated that these enzymes affect proliferation negatively and act in myogenic differentiation. We subsequently showed that both PRMT5 and CARM1 control fast muscle fiber formation whereas PRMT5 triggers slow muscle fiber formation. Finally, we found that if a decrease of CARM1 expression was not affecting the initial step of slow muscle fiber formation it lead to a defect in their localization. Hence, our results indicate that PRMT5 and CARM1 play a combinatorial role in zebrafish myogenesis.

## Results and Discussion

### *CARM1* and *PRMT5* are dynamically expressed during development

**Figure 1:**
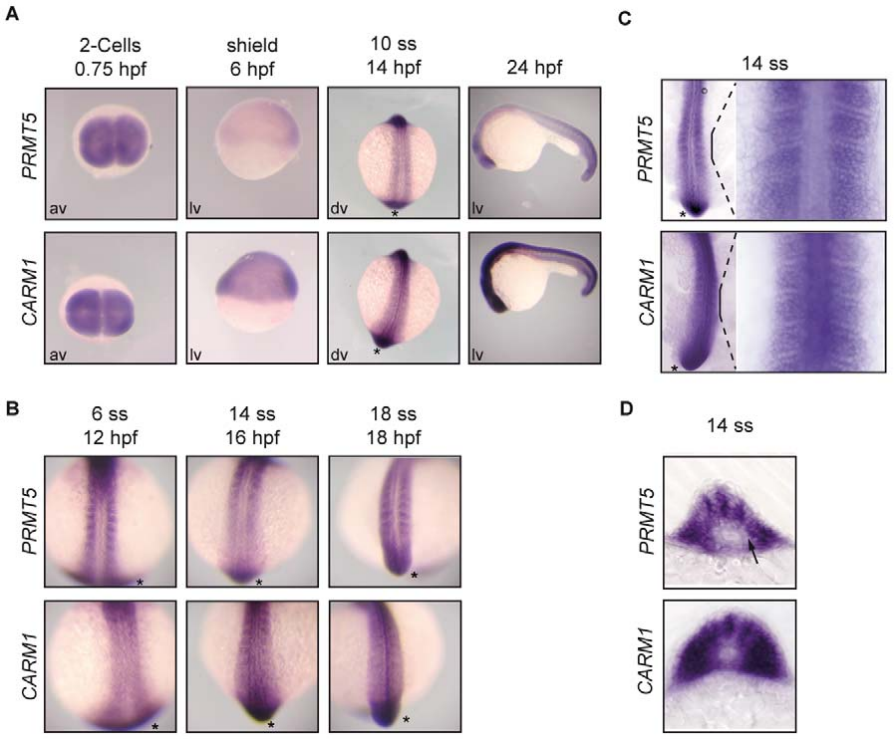
*PRMT5* and *CARM1* are differentially expressed during zebrafish embryogenesis. (A–D) *CARM1* and *PRMT5 in situ* hybridization at the indicated stages. (A) Animal view (av), lateral view (lv, dorsal to right for 6 hpf, anterior to left for 24 hpf) and dorsal views (dv, anterior to top) of whole-mount zebrafish *in situ* hybridization. (B) Dorsal view (anterior to top) of *CARM1* and *PRMT5* expression during somitogenesis. (C) Dorsal flat-mounts of 14 ss embryos stained for *CARM1* and *PRMT5*. A magnified region is shown on the right. (D) Transverse sections (30 µm) of the magnified region in (C) showing undetectable *PRMT5* expression in adaxial cells (arrow), whereas *CARM1* is ubiquitously expressed in the somite. *, Presomitic Mesoderm (PSM); ss, somite stage.

Expression pattern of *CARM1* and *PRMT5* during zebrafish development was first analyzed by *in situ* hybridization. We found that both *CARM1* and *PRMT5* were robustly expressed at 2-cell stage indicating that they are maternal genes (Figure 1A). At gastrulation, *CARM1* was strongly expressed in the dorsal shield, whereas *PRMT5* was weakly present in this domain. Of note, both *CARM1* and *PRMT5* were expressed within the somites during early somitogenesis and enriched in the presomitic mesoderm (PSM, Figure 1A–1C, asterisk), a structure that will give rise to the somites [19]. That both genes are expressed in the PSM suggests that CARM1 and PRMT5 may contribute to the maintenance of somite progenitors in this area.

Interestingly, *PRMT5* and *CARM1* were expressed in the somites from 6- to 18-somite stage during segmentation (Figure 1A and 1B). Around 24 hours post fertilization (24 hpf), somitogenesis is completed and *PRMT5* was preferentially expressed in the posterior trunk and in tail somites with a weaker expression in anterior trunk somites, whereas *CARM1* was expressed all along the axis with a strong expression in the anterior neural structures (Figure 1A). To gain further insight into the expression of *CARM1* and *PRMT5* in the somites, we analyzed them in flat-mounted embryos and in the corresponding cross sections at 14-somite stage (14 ss). *PRMT5* expression was not detected in the adaxial progenitor cells and was enriched in the medial part of the somite, whereas *CARM1* was ubiquitously expressed in the somite (Figure 1C and 1D). That *PRMT5* and *CARM1* are expressed in different parts of the somites suggests that they may act differentially on myogenesis.

### CARM1 and PRMT5 are essential for proper myogenesis

**Figure 2:**
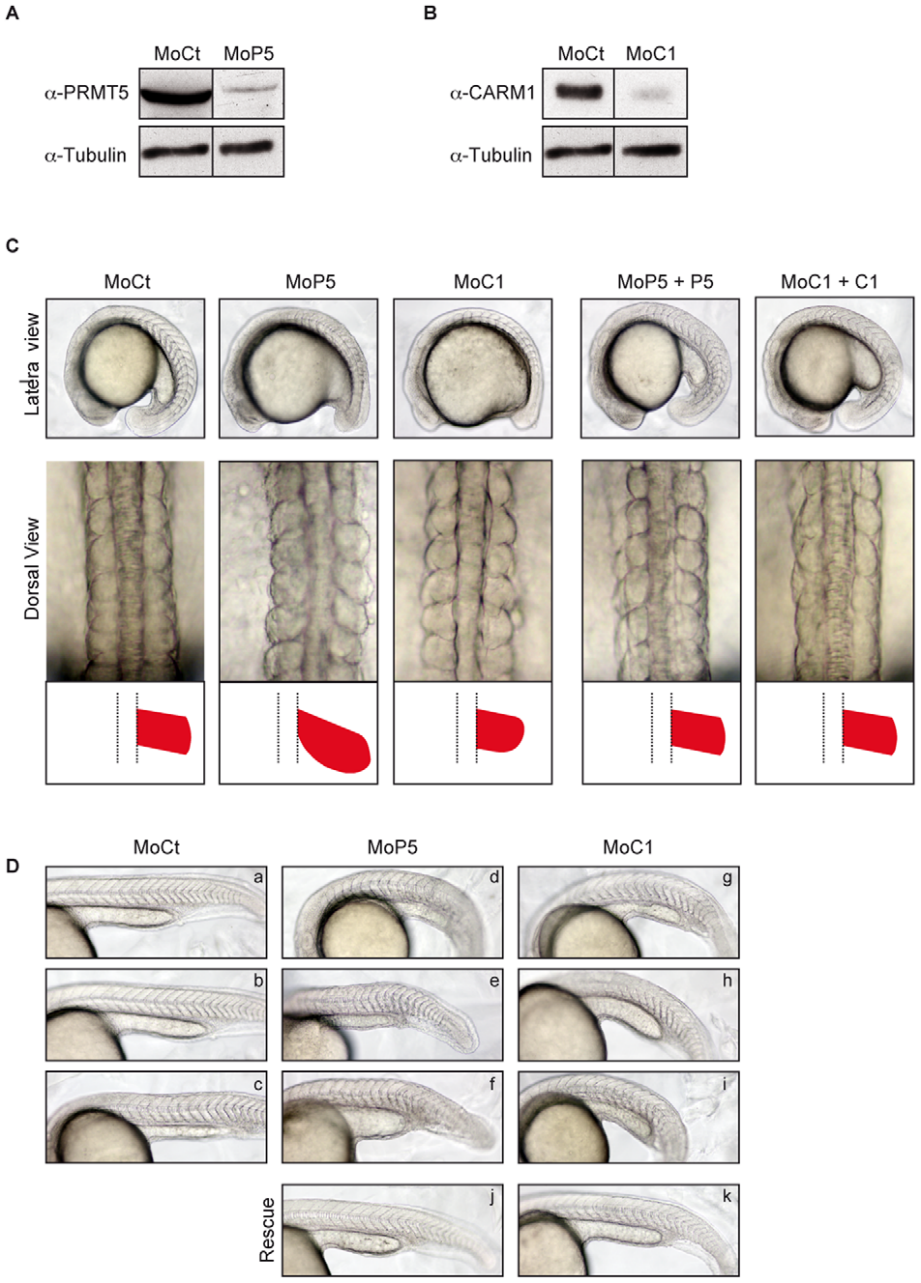
PRMT5 and CARM1 morphants exhibit distinct and specific phenotypes. (A, B) One-cell embryos were injected with 6 ng of either a control morpholino (MoCt), or a morpholino against PRMT5 (MoP5) or CARM1 (MoC1). Embryos were collected at 14 ss and were processed for immunoblotting to detect (A) PRMT5 and (B) CARM1 expression. Tubulin was used as a loading control. (C) Lateral (anterior to left) and dorsal view (anterior to top) of 14–16 ss zebrafish embryos injected with the indicated morpholino. Rescue experiments by injection of the cognate mRNA are shown to the right (MoP5+P5, MoC1+C1). Schematic representations of each somite are shown below the corresponding dorsal view. Mo, morpholino. (D) Phenotypes of 24 hpf embryos injected with increasing doses (3-6-12 ng) of Control (MoC, a–c), PRMT5 (MoP5, d–f) or CARM1 (MoC1, g–i) morpholinos or 6 ng of morpholino against PRMT5 or CARM1 co-injected with their corresponding mRNA (MoP5+P5, j and MoC1+C1, k) for rescue experiment. Embryos were visualized at 24 hpf. Lateral view, anterior to the left.

To examine the involvement of CARM1 and PRMT5 in myogenesis, we interfered with their translation using antisense morpholinos (Mo). Expression of endogenous CARM1 and PRMT5 was strongly reduced after injection of their respective morpholino, as confirmed by western blot (Figure 2A and 2B). Phenotypes of the morphants on the somites were analyzed at 14–16 ss and 24 hpf under polarized light. PRMT5 loss of function gave rise to embryos with long and flat somites, whereas CARM1 morphants showed smaller and rounder somites (Figure 2C and Table 1). Of note, the same morphant phenotypes were obtained after injection of another specific morpholino against *CARM1* or *PRMT5* (data not shown). These phenotypes were rescued at both stages when *CARM1* or *PRMT5* mRNAs were co-injected with their cognate morpholino (Table 1, Figure 2C, panels MoP5+P5, MOC1+C1 and Figure 2D, panels j and k). Furthermore, the phenotypes observed at 24 hpf were dependent on the dose of morpholinos injected (Table 1 and Figure 2D). Altogether, these data demonstrate the specificity of CARM1 and PRMT5 morpholinos and indicate that both CARM1 and PRMT5 are required for proper axis formation. The round shape of the somites at 14 ss in CARM1 morphant suggests that CARM1 could be involved in the elongation of muscle progenitor cells that are initially round to generate the myotome, a transition stage involved in somite boundary formation [20].

**Table 1:** Morphological defects of somites in zebrafish embryos injected with morpholinos against CARM1 or PRMT5.

	Percentage of embryos for each class of phenotype			
Number of embryos injected (n)	Wild type	Low	Mild	Severe
**14 ss**				
MoCt (465)	100	0	0	0
MoC1 (355)	35	65*	0	0
MoP5 (431)	0.07	0	99.93#	0
MoC1+C1 (370)	87.5	12.5*	0	0
MoP5+P5 (295)	90	0	10#	0
**24 hpf**				
MoCt (111)	100	0	0	0
MoC1a (142)	28.5	71.5	0	0
MoC1b (116)	0	22.4	77.6	0
MoC1c (129)	0	29.5	24	46.5**
MoCt (136)	100	0	0	0
MoP5a (126)	0.02	0	99.98	0
MoP5b (119)	0	0	45.3	54.7**
MoP5c (96)	0	0	19.8	80.2**

14 ss: embryos were injected at the one cell stage with 6 ng of morpholinos against CARM1 (MoC1), PRMT5 (MoP5) or control morpholinos (MoCt). Rescue experiments were performed by co-injecting MoC1 or MoP5 with the mRNA coding for *CARM1* (MoC1+C1) or *PRMT5* (MoP5+P5), respectively.

24 hpf: Morpholino doses used for 24 hpf phenotype analysis were the following: 3 ng (a), 6 ng (b) and 12 ng (c). Percentages of embryos exhibiting the various phenotypes at 14 ss and 24 hpf are shown.

*Round, smaller somites;

# Flat, longer somites;

**Shorter axis.

### PRMT5 and CARM1 regulate differentially myogenic factor expression

To further characterize the impact of CARM1- and PRMT5-loss of function, we first focused on their putative role on mesodermal markers expression at gastrulation. We found that although *CARM1* and *PRMT5* were expressed in the shield during gastrulation (Figure 1A), their knock down did not affect the expression of the organizer gene *goosecoid/gsc* (data not shown), or of the mesodermal gene *spadetail/spt/tbx16* (Figure S1A), a key regulator of *myod* expression [21], [22]. Hence, CARM1 and PRMT5 are not required in early mesoderm specification, from where the somites arise.

**Figure 3:**
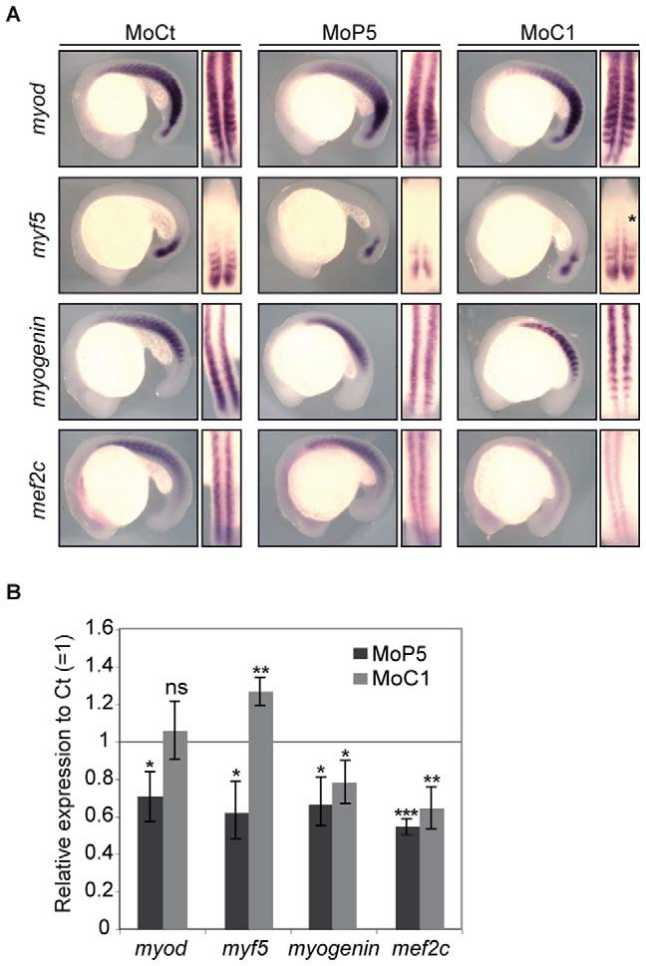
PRMT5 and CARM1 regulate myogenic factor expression. Expression of *myod*, *myf5*, *myogenin* and *mef2c* at 18 ss in embryos injected with the indicated Mo by (A) whole-mount *in situ* hybridization (lateral view, anterior to left; a dorsal view, anterior to top, is shown on the right; *, anterior expression of *myf5* in CARM1 morphant) or (B) real time PCR. Error bars represent standard deviations. *, P<0.01 statistically significant; **, P<0.001, very statistically significant; ***, P<0.0001, extremely statistically significant; ns, not statistically significant, using a t-test.

We then analyzed the impact of CARM1- or PRMT5- knock down on the expression of key regulators of zebrafish myogenesis, *myod*, *myf5*, *myogenin* and *mef2c* at 14 ss (Figure S1B and Figure 2) and at 18 ss (Figure 3). Strikingly, we observed that at 14 ss PRMT5 Mo diminished the expression of *myod*, whereas CARM1 knock down had no effect on the expression of this gene (Figure S1B). In addition, PRMT5 morphants exhibited a significant reduction of *myf5*, *myogenin* and *mef2c* expression (Figure 3 and Figure S2). *Myf5* and *mef2c* expression were affected all along the axis, whereas *myogenin* expression was mostly diminished in the posterior somites, that host the fast muscle precursors (Figure S2B, arrow), and faintly decreased in the medial part of the somite. On other hand, CARM1 depletion down-regulated the expression of *myogenin* and *mef2c* (Figure 3 and Figure S2B) and lead to a slight increase of *myf5* expression in the most medial part of anterior somite (Figure 3A asterisk, and Figure S2A asterisk). To confirm these results, we analyzed the expression of these genes by quantitative PCR on Mo-injected 14 ss (Figure S2B) and 18 ss (Figure 3B) embryos. Consistent with the above results, PRMT5 knock down induced a significant decrease of *myf5, myogenin* and *mef2c* expression levels whereas CARM1 knock down lead to a decrease of *myogenin* and *mef2c* levels and to an increase of *myf5* expression at 18 ss (Figure 3B and Figure S2B). Of note, PRMT5 or CARM1 loss did not appear to affect the expression of each other (Figure S3), suggesting that the phenotypes observed in each morphant were not due to the deregulation of the other enzyme. Accordingly, decrease of *myogenin* expression by either PRMT5 or CARM1 Mo was specifically and strictly rescued by the cognate mRNA and not by the other one (Figure S4). Furthermore, that myogenic gene expression was affected in a similar way in the morphants at 18 ss as at 14 ss further supports that the effects observed are specific and excludes any developmental delay (Figure 3 and Figure S2).

It has been shown previously that PRMT5 was required for the activation of *myogenin* in cell culture and in muscle tissues [4], [5]. Our study reveals that PRMT5 controls myogenesis at an earlier step by regulating *myod* and *myf5* expression. Hence, it is likely that PRMT5 controls myogenesis *via* a two-tiers mechanism: first, our study shows that it up-regulates *myod* (Figure 3 and Figure S1B) and *myf5* expression (Figure 3 and Figure S2), second, as demonstrated in cell culture, it also cooperates with Myod or Myf5 to activate *myogenin* expression [5]. As PRMT5 morphant did not show any defect in *spadetail* expression (Figure S1A), a gene that controls the onset of *myod* expression [21], [22], we propose that PRMT5 may activate directly *myod* and *myf5* expression by being recruited onto their promoters as it has been demonstrated for *myogenin* in cell-based assays [4]. However, we cannot rule out that PRMT5 could also affect their expression indirectly or post-transcriptionnally *via* its enzymatic activity.

Interestingly, our data reveal that CARM1 is required to specifically control the down-regulation of *myf5* expression in the mature somite (Figure 3). It has been shown that CARM1 methylates RNA-binding protein HuD thereby affecting the stability of HuD target mRNAs [23] and that CARM1 can act either positively or negatively on mRNA stability of a certain number of c-Fos target genes [24]. One can speculate that CARM1 could regulate *myf5* expression post-transcriptionnally in a similar way. Our data showing that CARM1 regulates *myogenin* expression *in vivo* are in agreement with the original report showing that CARM1 activates *myogenin* expression in cell-based studies [3]. However, a recent study has reported a role of co-activator of Myogenin-dependent transcription for CARM1 but without affecting *myogenin* expression [5]. Of note, this study was performed in Myod-differentiated fibroblasts and C2C12 myoblasts. Here, our data indicate that in a whole organism, CARM1 regulates *myf5*, *myogenin* and *mef2c* expression. Hence, PRMT5 and CARM1 are not only co-activators of Myod- and Myogenin-dependent transcription respectively, but they also control the expression of these myogenic factors.

### PRMT5 and CARM1 control slow and fast myogenesis differentially

**Figure 4:**
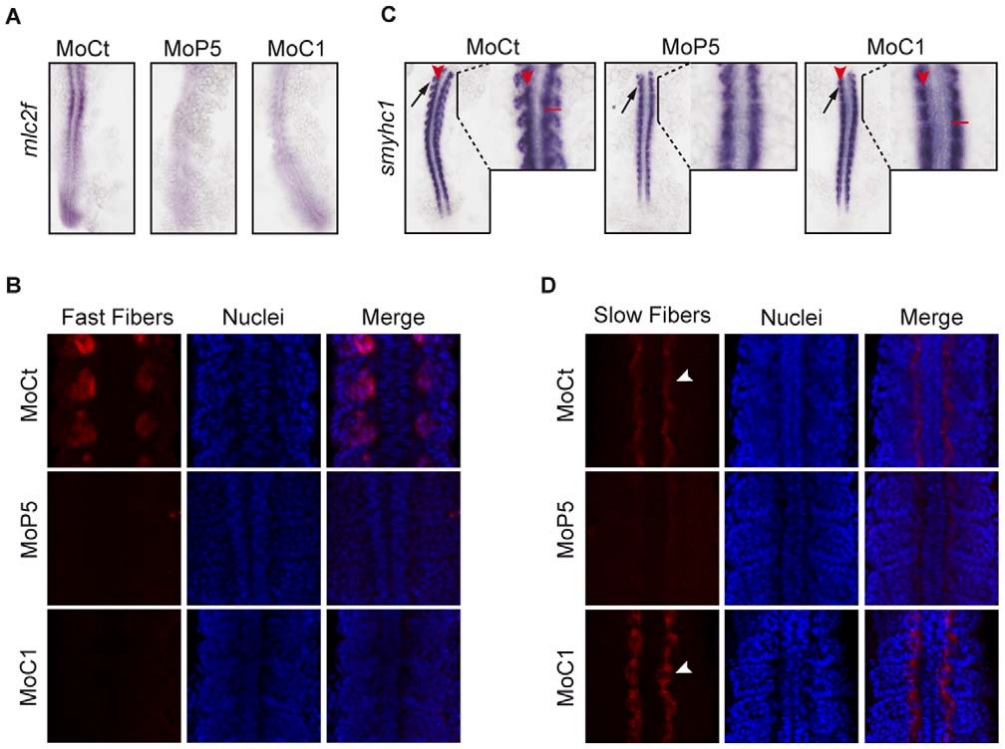
CARM1 and PRMT5 differentially control slow and fast myogenesis. (A, C) Dorsal flat-mounted 14 ss embryos (anterior to top) injected with the indicated Mo were analyzed by whole-mount *in situ* hybridization for (A) *mlc2f* and (C) *smyhc1* or (B, D) immunohistochemistry for (B) fast fibers, (D) slow fibers. In (C) a magnification of the area indicated by the black line is shown to the right of each panel. Arrow or white arrowhead, lateral expression; red arrowhead, medial expression; red bar, length of one somite. (B, D) Z-stack sections of the most anterior somites are shown.

To address whether PRMT5 and CARM1 could affect slow and/or fast myogenesis we analyzed the expression of *slow myosin heavy chain 1 (smyhc1)*, a slow fiber specific gene and of *fast myosin light chain* (*mlc2f*), a fast fiber specific gene in PRMT5- and CARM1-depleted embryos as compared to control embryos. In PRMT5 morphants, *mlc2f* was dramatically reduced as compared to control morphants (Figure 4A) and *smyhc1* expression was completely lost in the lateral part of the somite (Figure 4C, arrow). CARM1 knock down restrained *smyhc1* expression medially (Figure 4C, red arrowhead) and *mlc2f* expression almost disappeared (Figure 4A). These data were confirmed by immunohistochemistry against fiber-specific myosin isoforms at two different stages (14 ss, Figure 4B and 4D; 18 ss, Figure S5). PRMT5 knock down lead to a strong decrease in slow muscle fiber formation and suppressed fast muscle fibers at both stages (Figure 4B and 4D and Figure S5).

Interestingly, *PRMT5* was not detected in adaxial cells (Figure 1D) and our results suggest that this enzyme can control slow muscle fiber formation. Several possibilities can be proposed to explain this apparent paradox. First, even though PRMT5 was not detected in adaxial cells in our *in situ* hybridization experiments, it could be nevertheless very weakly expressed and thus active in these cells. Another possibility is that PRMT5 could act non-cell autonomously in adaxial cells. Finally, PRMT5 would not be required for the activation of *smyhc1* expression but for its maintenance. Indeed, if expression of *smyhc1* was weakly affected by PRMT5 knock down, its protein level was barely detectable in the same condition (Figure 4C and 4D). These results suggest that PRMT5 controls *smyhc1* expression post-transcriptionally. We can speculate that, either PRMT5 is specifically required for *smyhc1* translation, or PRMT5 is somehow involved in SMYHC1 stability. Interestingly, it has been shown recently that PRMT5 was required for the expression of myogenic microRNAs [25]. Hence, one possibility is that PRMT5 could control *smyhc1* translation by affecting the expression of these miRNAs. Further studies would be needed to elucidate whether and how PRMT5 controls SMYHC1 protein synthesis and/or stability.

**Figure 5:**
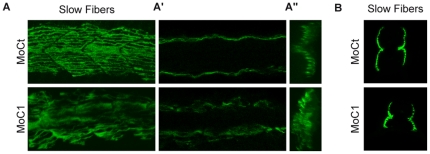
CARM1 controls slow fiber localization. (A, B) One-cell stage embryos were injected with a CARM1 morpholino (MoC1) or a control morpholino (MoCt) and were analyzed at 24 hpf by whole-mount immunohistochemistry for slow fibers. (A) Lateral views, anterior to the left of 3D confocal reconstructions projected over four somites, (A′) single confocal scans at the dorsoventral level of the notochord and (A″) corresponding cross-section views. (B) Rostral cross-sections (50 µm) of 22 ss embryos injected with the indicated morpholino.

CARM1 morphants exhibited a lack of fast muscle fiber formation and a restriction of the slow fiber marker to the midline (Figure 4B and 4D and Figure S5). This pattern is reminiscent of embryos mutated in *M-* or *N- Cadherin* that control slow muscle cell migration in the developing zebrafish myotome [26] and is in line with CARM1 morphant phenotype that generates round somites. We then went on to analyze slow fiber proper localization in CARM1 knock down. We found that slow muscle fiber localization was affected in CARM1 morphants (Figure 4D and Figure 5). More investigations will be needed to analyze whether CARM1 is involved in slow muscle fiber localization and regulates *cadherin* expression. Altogether, these results clearly show that PRMT5 is essential for slow and fast myogenesis, whereas CARM1 only controls fast fiber formation and is required for proper slow muscle fiber localization.

**Figure 6:**
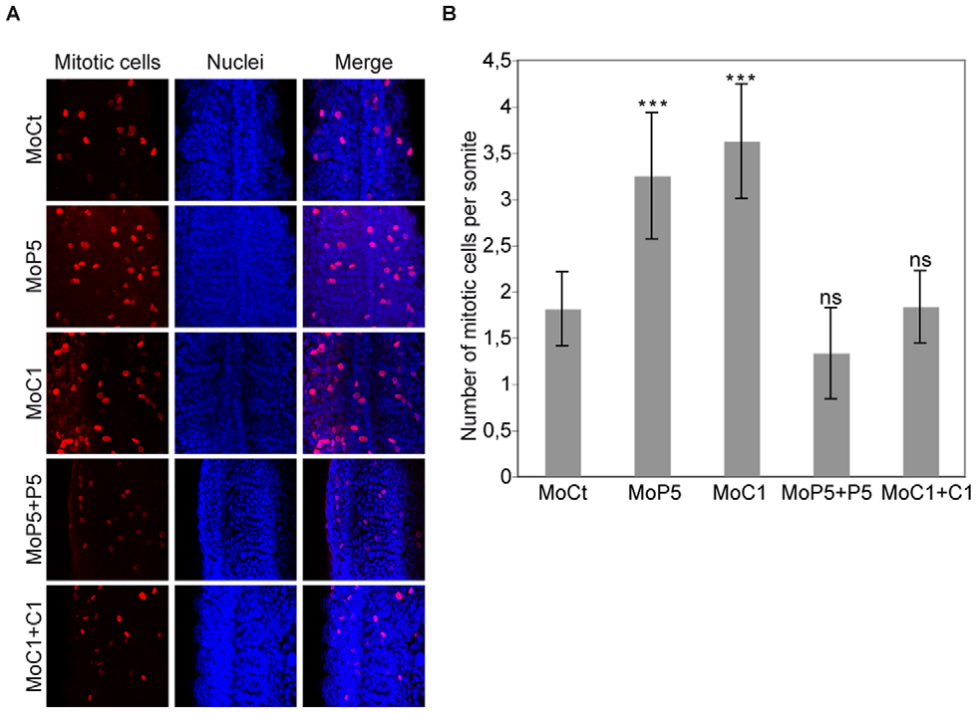
CARM1 and PRMT5 control cell cycle arrest during development. (A) Dorsal flat-mounted of 14 ss embryos (anterior top) injected with the indicated Mo or co-injected with the corresponding mRNA for rescue experiments were analyzed by immunohistochemistry for mitotic cells with an anti phospho-H3 (Ser10) antibody. (B) Representation of the number of mitotic cells per somite. Error bars represent standard deviations. ***, P<0.0001 extremely statistically significant; ns, not statistically significant, using a t-test.

Having shown that CARM1 and PRMT5 control muscle cell fiber formation, we analyzed whether they also regulate cell proliferation in the somite as CARM1 and PRMT5 have been shown in cell culture to regulate cell proliferation, either positively [27] or negatively [14], respectively. Surprisingly, we found that at 14 ss, loss of either CARM1 or PRMT5 increased significantly the number of mitotic cells within the somite (Figure 6). This effect was rescued when *CARM1* or *PRMT5* mRNAs were co-injected with their cognate morpholino (Figure 6, MoP5+P5, MoC1+C1). Hence, these data indicate that both enzymes trigger cell cycle arrest and myogenic differentiation during development. We propose that CARM1 and PRMT5 could control the timing of muscle fiber formation by regulating the switch between proliferation and cell cycle exit.

Here, we have established a combined action of CARM1 and PRMT5 to control *MRF* and *mef2c* expression as well as slow and fast myogenesis in zebrafish. Recent studies have emphasized the role of methylation of non histone proteins such as transcriptional co-regulators, transcription elongation factors and RNA binding proteins by CARM1 and PRMT5 [28]. It is tempting to speculate that through their enzymatic activity on specific substrates, these enzymes could regulate the transcriptional activity, protein-protein interactions, protein stability and/or protein sub-cellular localization of key factors involved in myogenesis. It was found that CARM1 interacts with Myogenin and Mef2D, whereas PRMT5 can bind Myod, Myogenin and Mef2D *in vitro* [5]. To further elucidate how these enzymes control myogenesis it would be of particular interest to analyze whether they methylate some myogenic factors. Indeed, methylation could modulate the interactions between MRFs or Mef2 and specific partner(s), notably transcriptional co-activators/mediators or ATP-dependent chromatin remodeling enzymes to activate transcription on target genes.

In summary, this study demonstrates that CARM1 and PRMT5, two transcriptional co-regulators that have been shown respectively to act positively and negatively on proliferation in cell-based assays, are both inhibiting proliferation and are required for myogenic differentiation in a whole organism. However, they play distinct roles in myogenesis: PRMT5 regulates slow and fast fiber formation by controlling early myogenic genes (*myod* and *myf5*) as well as *myogenin*, whereas CARM1 is required for fast fiber formation by regulating *myogenin* expression. Interestingly, our data also show that if CARM1 has no impact on slow fiber formation it is required for their proper localization.

## Methods

### Ethics Statement

All embryos were handled according to relevant national and international guidelines. French Ministery of Agriculture approved the protocols in this study, with approval ID: B-31-555-10.

### Embryos, synthetic mRNAs, morpholinos and injections

Zebrafish were raised according to standard procedures [29]. Injections were performed at one-cell stage with 250 pg of *CARM1* RNA [6], 250 pg of *PRMT5* RNA [30] and 6 ng of the indicated morpholinos (Gene-tools). Morpholino sequences were: PRMT5 5′-GACGCCATCGTTAGGAGACGAGATG-3′; CARM1 5′-GAGAACACGGACACCGC CATCTTCG-3′; Control 5′-CCTCTTACCTCAG TTACAATTTATA-3′.

### Whole-mount in situ hybridization, immunostaining and image acquisition

Whole-mount *in situ* hybridization and antibody staining were performed according to standard protocols. *In situ* hybridization was performed with digoxigenin-labeled probes transcribed from plasmids containing cDNA for *spt* [31], *myod* and *myogenin* [22], *myf5* [32], *mef2c* [33], *smyhc1* [34] and *mlc2f* [35]. *zCARM1* and *zPRMT5* anti-sense probes were obtained by sub-cloning their corresponding ESTs BC078292 and BC095362 respectively (Open Biosystems, Huntsville, AL, US), into pBluescript KS (Stratagene, La Jolla, CA, US) using EcoRI/HindII (zCARM1) and XmaI/HindIII (zPRMT5) and were transcribed with T3 polymerase. For cross-sections, embryos were embedded in 3% Low Melting Agar after whole-mount *in situ* hybridization and 30 µm sections were performed using a Leica vibratome (VT 1000S). For immunohistochemistry, the following antibodies were used: F310 fast Myosin Light Chain (DSHB), F59 slow Myosin Heavy Chain (DSHB) and phospho-Histone H3 (ser10) (Upstate, Greenville, SC, US) with appropriate Alexa Fluor-conjugated secondary antibodies (Molecular Probes, Eugene, OR, US). Nuclei were stained with TO-PRO3 (Molecular Probes, Eugene, OR, US) according to the manufacturer's protocol. Embryos were dissected, flat-mounted in glycerol or mounted in Mowiol and images were recorded on a microscope (NIKON Eclipse 80i) using a 20× Plan Apo na 0.5 or a 40× plan Apo na 1 with the NIS-element AR 2.30 software; or on a confocal microscope (TCS SP5, Leica Microsystems) with a 20× Plan Apo na 0.7 objective (zoom ×4) using the scanner resonant mode. Confocal images are stacks of the anterior somites of 14 or 18 ss embryos as stated in figure legends. For whole embryos, imaging was performed using a stereomicroscope (Leica MZ FL III) with the ACT-1C software.

### RNA extraction and reverse transcription

Total RNAs were extracted from 25 embryos at 14 ss or 18 ss as stated in figure legends with the RNeasy mini kit (Qiagen, Valencia, CA, US), eluted in RNAse free water and reverse-transcribed with the Superscript II Reverse Transcriptase (Invitrogen, Carlsbad, CA, US) according to the supplier's instructions.

### Quantitative PCR

Q-PCR analyzes were performed on an i-Cycler device (Biorad, Hercules, CA, US) with the SYBR green JumpStart Taq Ready mix (Sigma Aldrich, St. Louis, MO, US), according to the manufacturer's instructions. All experiments included a standard curve. Samples were analyzed in triplicates and the mean and standard deviation were calculated. Samples were normalized to the number of *EF1* mRNA copies. Primer sequences were the following: *myod* fw 5′- GCCCAAAGTGGAGATTCTGA-3′, rev 5′- GCCCATAAAATCCATCATGC-3′; *myf5* fw 5′-GAGAGCATGGTTGACTGCAA-3′, rev 5′-GAATCACTTCCGGTTGGAGA-3′; *myogenin* fw 5′-AAACCATCTCCATCGTCCAG-3′; rev 5′-GGGTTCATCAATGT GCTCCT-3′; *Mef2c* fw 5′-GGTCTCTCCAGGGAACATGA-3′, rev 5′-GCCCATCACTT CTCCAGGTA-3′; *EF1-alpha* fw 5′-GATGCACCACGAGTCTCTGA-3′, rev 5′-TGATGACCT GAGCGTTGAAG-3′.

### Western Blotting

Whole cell extracts from 25 zebrafish embryos were classically prepared in 50 µl Laemmli sample buffer. 10 µl of each sample (5 embryos) were loaded per lane and subjected to SDS-PAGE. The following commercial antibodies were used: anti-CARM1 (US Biologicals, P9004-20C), anti-PRMT5 (Upstate, 07-405), anti alpha-Tubulin (Sigma, T9026), anti-Rabbit IgG HRP conjugate (Promega, W4011) and anti-Mouse IgG HRP conjugate (Promega, W4021).

## Supporting Information

Figure S1 **PRMT5 and CARM1 regulate myogenic factor expression.** (A–B) *In situ* hybridization of embryos with the indicated mRNA probe and injected with the indicated Mo (A) at the shield stage or (B) at 14 ss. (A) Animal view (dorsal to right). (B) Dorsal flat-mounted embryos stained for *myod* with a magnified region to the right.
(TIF)

Figure S2 **PRMT5 and CARM1 regulate specifically myogenic factors expression at 14-somite stage (14 ss).** Whole-mount *in situ* hybridization of embryos injected at one-cell stage with the indicated morpholino (Mo). Experimentrs were done twice with n = 20 for each condition. Embryos were collected at 14 ss and were analyzed for myogenic factors expression by (A) *in situ* hybridization, lateral view, anterior to the left or by (B) real time PCR with standard deviations relative to a control morpholino. Q-PCR procedures are detailed in the methods section. (A) Asterisk, anterior expression of *myf5* in CARM1 morphant; arrow, down regulation of myogenin expression in the posterior somites in PRMT5 morphant. (B) Error bars represent standard deviations. *, P<0.01; **, P<0.001; ***, P<0.0001; ns, not statistically significant.
(TIF)

Figure S3 **Knock down of CARM1 or PRMT5 does not affect their mutual expression.** One-cell stage embryos injected with either a control morpholino (MoCt), or a morpholino against PRMT5 (MoP5) or against CARM1 (MoC1) (left panels). Mos were co-injected with either *PRMT5* mRNA (MoP5+P5) or *CARM1* (MoC1+C1) (right panels). Embryos were collected at 14-somite stage and analyzed for *CARM1* and *PRMT5* expression by *in situ* hybridization. Note that *PRMT5* expression is strongly enhanced in MoP5-injected embryos (white asterisk), which can be rescued by the co-expression of *PRMT5* mRNA (black asterisk).
(TIF)

Figure S4 ***PRMT5*** **and** ***CARM1*** **mRNAs rescue specifically** ***myogenin*** expression affected by their cognate morpholino(*). One-cell stage embryos were injected with either a morpholino control (MoCt), or a morpholino against PRMT5 (MoP5) or CARM1 (MoC1), alone or in combination with either *PRMT5* or *CARM1* mRNA. Embryos were collected at 14-somite stage and were analyzed for *myogenin* expression by *in situ* hybridization. Experiments were done twice (n = 22 for each condition).
(TIF)

Figure S5 **CARM1 (C1) and PRMT5 (P5) control myogenesis differentially.** (A,B) One-cell stage embryos were injected with the indicated morpholino (Mo) or a control Mo (MoCt) and were analyzed by whole-mount immunohistochemistry for (A) slow fibers and (B) fast fibers at 18-somite stage. Lateral views, anterior to the left. Both slow and fast fiber formation require PRMT5 (n = 12). CARM1 is necessary for fast fiber specification but does not affect slow fiber specification (n = 15). Antibodies used were: F310 fast Myosin Light Chain (DSHB), F59 slow Myosin Heavy Chain (DSHB) and appropriate Alexa Fluor-conjugated secondary antibodies (Molecular Probes, Eugene, OR, US). Nuclei were stained with TO-PRO3 (Molecular Probes, Eugene, OR, US) according to the manufacturer's protocol.
(TIF)
