# Supplementary figures and images for: The Methyltransferases PRMT4/CARM1 and PRMT5 Control Differentially Myogenesis in Zebrafish
Source: PLoS One. 2011 Oct 10;6(10):e25427. doi: 10.1371/journal.pone.0025427 (PMC3189919; doi:10.1371/journal.pone.0025427)

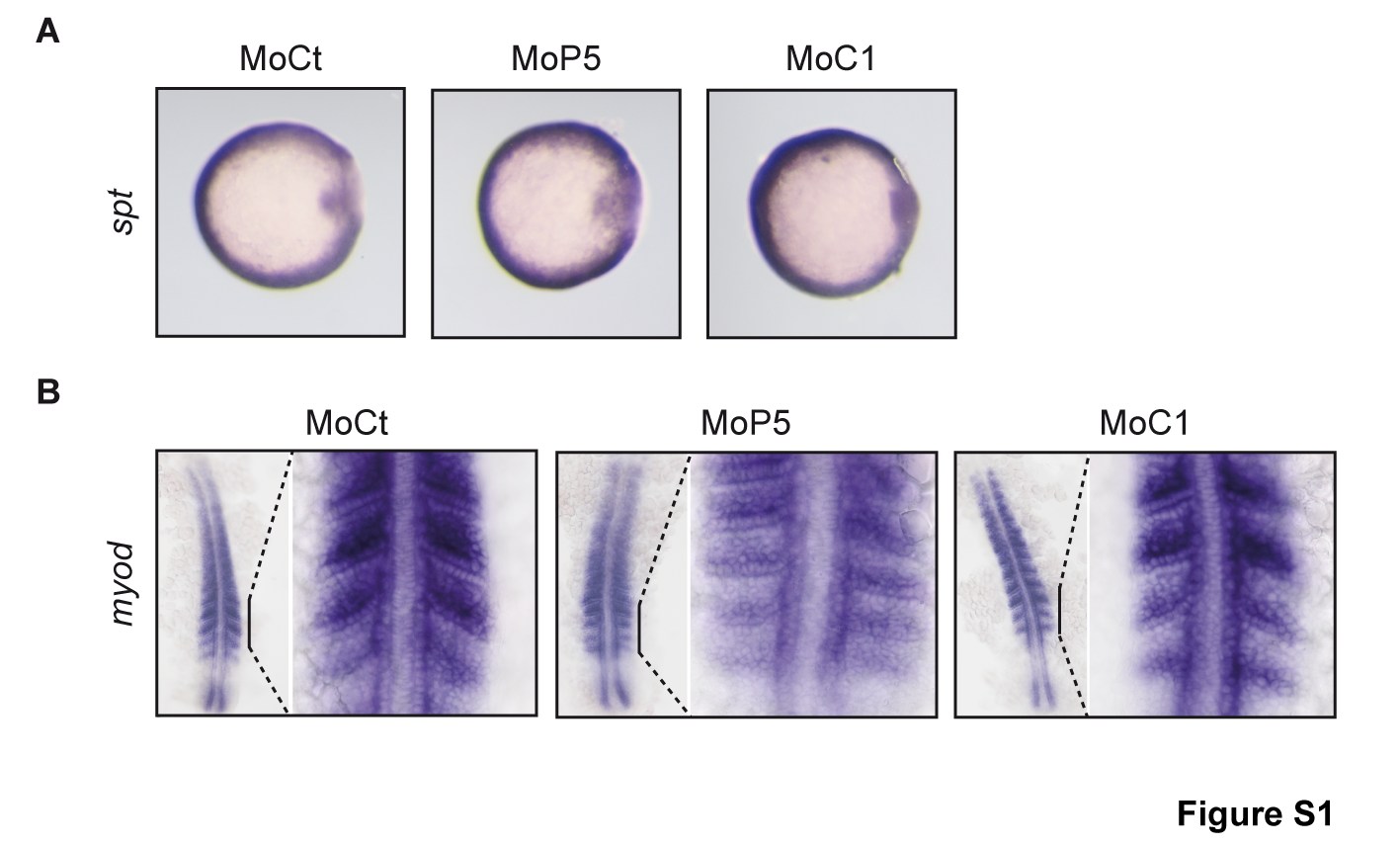

Supplement: Figure S1 — PRMT5 and CARM1 regulate myogenic factor expression. (A–B) In situ hybridization of embryos with the indicated mRNA probe and injected with the indicated Mo (A) at the shield stage or (B) at 14 ss. (A) Animal view (dorsal to right). (B) Dorsal flat-mounted embryos stained for myod with a magnified region to the right. (TIF) [file pone.0025427.s001.tif]

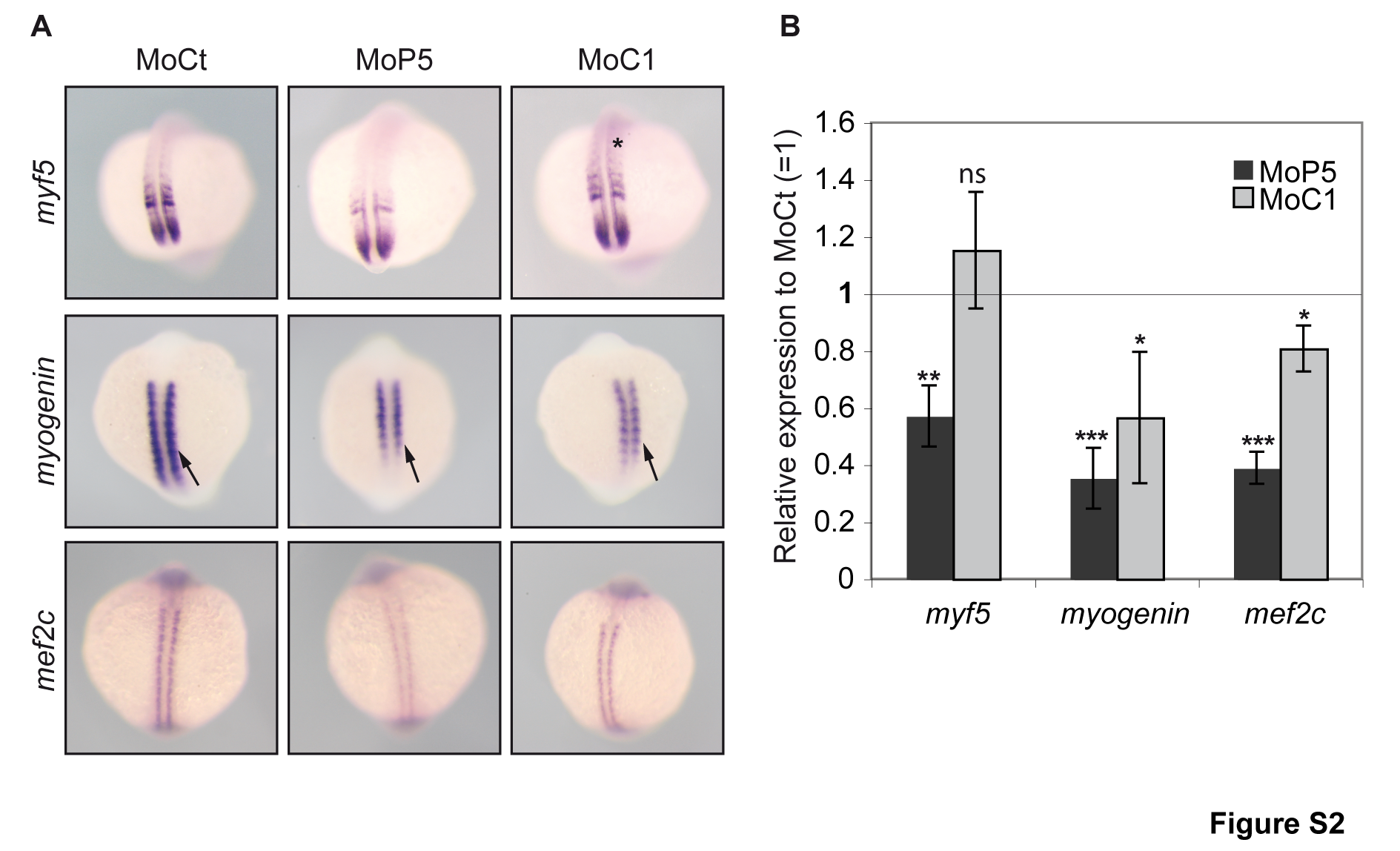

Supplement: Figure S2 — PRMT5 and CARM1 regulate specifically myogenic factors expression at 14-somite stage (14 ss). Whole-mount in situ hybridization of embryos injected at one-cell stage with the indicated morpholino (Mo). Experimentrs were done twice with n = 20 for each condition. Embryos were collected at 14 ss and were analyzed for myogenic factors expression by (A) in situ hybridization, lateral view, anterior to the left or by (B) real time PCR with standard deviations relative to a control morpholino. Q-PCR procedures are detailed in the methods section. (A) Asterisk, anterior expression of myf5 in CARM1 morphant; arrow, down regulation of myogenin expression in the posterior somites in PRMT5 morphant. (B) Error bars represent standard deviations. *, P<0.01; **, P<0.001; ***, P<0.0001; ns, not statistically significant. (TIF) [file pone.0025427.s002.tif]

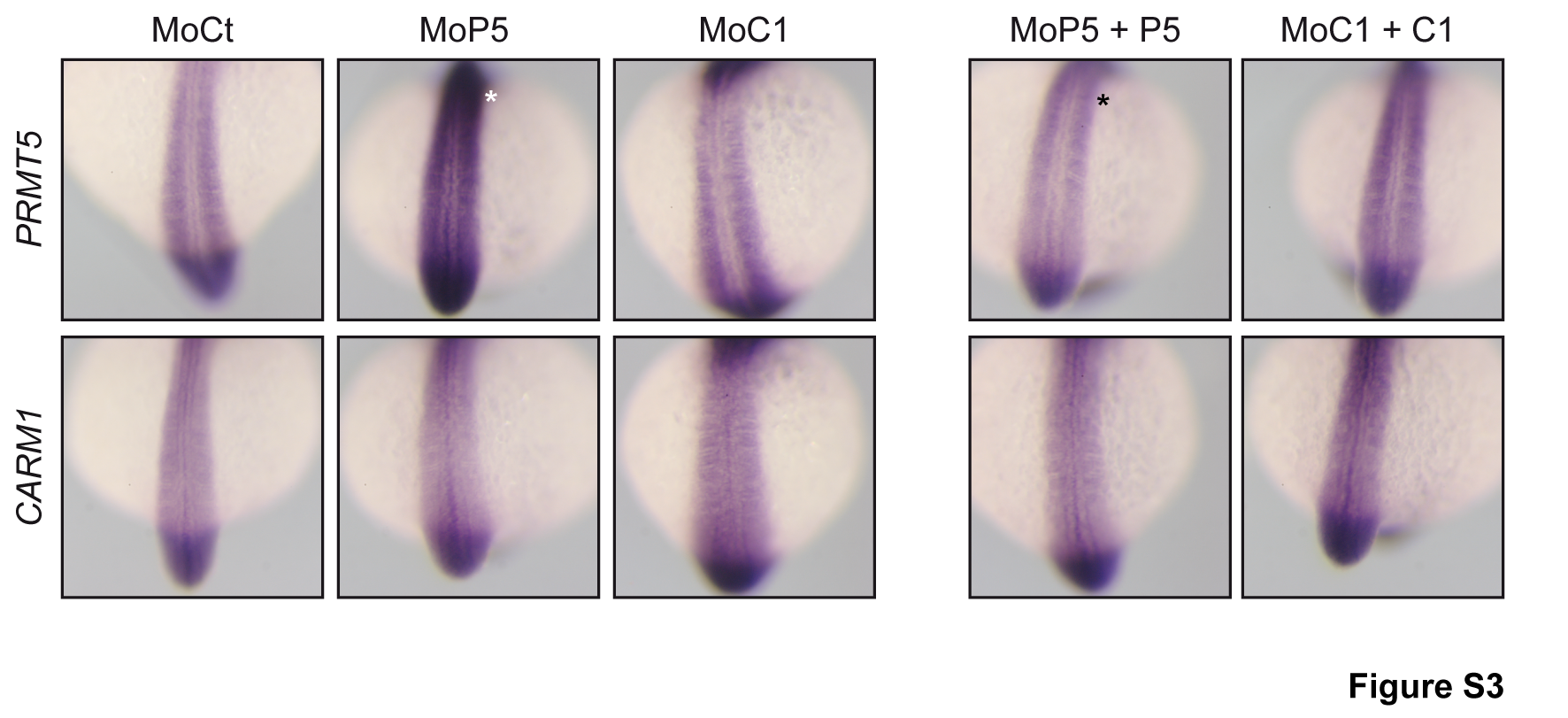

Supplement: Figure S3 — Knock down of CARM1 or PRMT5 does not affect their mutual expression. One-cell stage embryos injected with either a control morpholino (MoCt), or a morpholino against PRMT5 (MoP5) or against CARM1 (MoC1) (left panels). Mos were co-injected with either PRMT5 mRNA (MoP5+P5) or CARM1 (MoC1+C1) (right panels). Embryos were collected at 14-somite stage and analyzed for CARM1 and PRMT5 expression by in situ hybridization. Note that PRMT5 expression is strongly enhanced in MoP5-injected embryos (white asterisk), which can be rescued by the co-expression of PRMT5 mRNA (black asterisk). (TIF) [file pone.0025427.s003.tif]

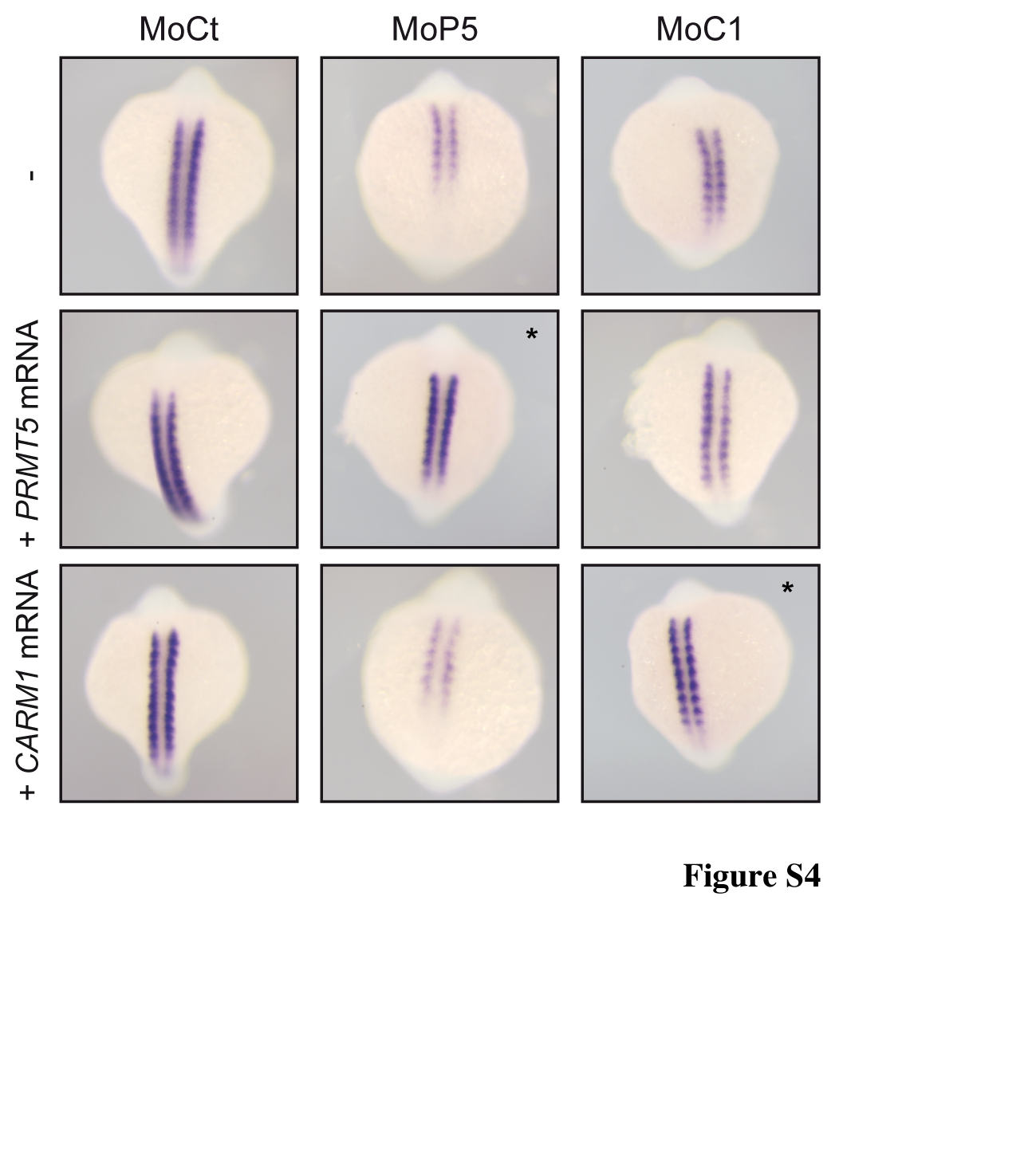

Supplement: Figure S4 — PRMT5 and CARM1 mRNAs rescue specifically myogenin expression affected by their cognate morpholino(*). One-cell stage embryos were injected with either a morpholino control (MoCt), or a morpholino against PRMT5 (MoP5) or CARM1 (MoC1), alone or in combination with either PRMT5 or CARM1 mRNA. Embryos were collected at 14-somite stage and were analyzed for myogenin expression by in situ hybridization. Experiments were done twice (n = 22 for each condition). (TIF) [file pone.0025427.s004.tif]

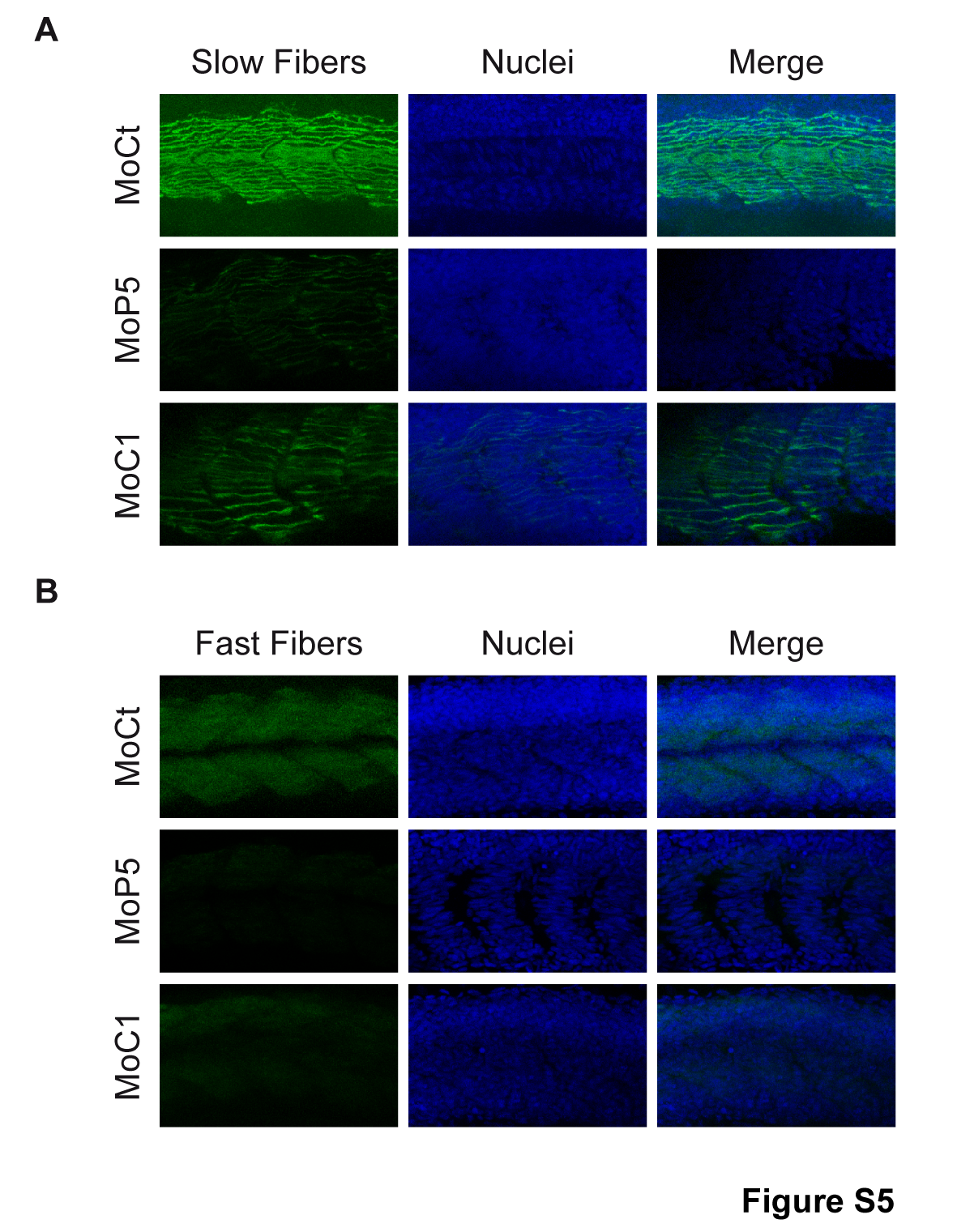

Supplement: Figure S5 — CARM1 (C1) and PRMT5 (P5) control myogenesis differentially. (A,B) One-cell stage embryos were injected with the indicated morpholino (Mo) or a control Mo (MoCt) and were analyzed by whole-mount immunohistochemistry for (A) slow fibers and (B) fast fibers at 18-somite stage. Lateral views, anterior to the left. Both slow and fast fiber formation require PRMT5 (n = 12). CARM1 is necessary for fast fiber specification but does not affect slow fiber specification (n = 15). Antibodies used were: F310 fast Myosin Light Chain (DSHB), F59 slow Myosin Heavy Chain (DSHB) and appropriate Alexa Fluor-conjugated secondary antibodies (Molecular Probes, Eugene, OR, US). Nuclei were stained with TO-PRO3 (Molecular Probes, Eugene, OR, US) according to the manufacturer's protocol. (TIF) [file pone.0025427.s005.tif]
